# Tumour distribution and characteristics associated with poor surgical outcomes in patients with sporadic spinal schwannomas

**DOI:** 10.1007/s00701-025-06439-1

**Published:** 2025-02-04

**Authors:** Shinsuke Yoshida, Takaaki Suzuki, Masayuki Tanabe, Kazuo Saita

**Affiliations:** 1 Department of Neurosurgery, Saitama Medical Centre, Saitama Medical University, Saitama, Japan; 2 Department of Orthopaedic surgery, Saitama Medical Centre, Saitama Medical University, Saitama, Japan

**Keywords:** Spinal schwannomas, Thoracic spine, Dumbbell-shaped tumours, Functional outcomes, McCormick

## Abstract

**Purpose:**

Spinal schwannomas are benign tumours that can compress the spinal cord or nerve roots, causing neurological symptoms. Despite successful surgical resection, some patients experience suboptimal functional recovery. Several risk factors for poor prognosis have been identified, but limited research has explored the influence of tumour distribution and characteristics. In this study, we aimed to identify prognostic variables associated with residual neurological deficit in patients undergoing surgical resection for sporadic spinal schwannomas.

**Methods:**

Clinical and radiological data of consecutive patients who underwent surgery for spinal schwannomas at Saitama Medical Centre between January 2010 and March 2024 were retrospectively reviewed. Patients with neurofibromatosis type 2 or foraminal and paravertebral schwannomas were excluded. Data collected included patient demographics, radiological features, and surgical complications. Residual neurological deficit was defined as a Modified McCormick scale grade of II–V, persistent neurogenic pain, or bladder/bowel dysfunction.

**Results:**

Gross total resection was achieved in 55 cases (76.4%). Postoperative complications occurred in 6 cases (8.3%), including cerebrospinal fluid fistula and vascular injury. At a median follow-up of 26.4 months, 20 patients (27.8%) had residual neurological deficits. Univariable and multivariable logistic regression identified thoracic spine involvement (odds ratio [OR], 5.03; 95% confidence interval [CI], 1.47–18.6; *p* = 0.01) and dumbbell-shaped tumours (OR, 0.15; 95% CI, 0.02–1.28; *p* = 0.04) as significantly associated with residual neurological deficits. Moreover, thoracic spinal schwannomas were associated with a significantly higher incidence of persistent postoperative neurogenic pain than that associated with cervical or lumbosacral tumours (*p* = 0.001).

**Conclusions:**

Thoracic spine involvement and tumours that are not dumbbell-shaped were identified as significant risk factors for residual neurological deficits in patients undergoing surgical treatment for spinal schwannomas. Awareness of tumour distribution and characteristics may assist in refining preoperative assessments, guiding strategic decisions, and potentially improving surgical management for better patient care.

## Introduction

Spinal schwannomas are benign, well-circumscribed tumours that can cause significant neurological deficits and pain due to spinal cord or nerve root compression. Surgical resection remains the first-line treatment for symptomatic spinal schwannomas. The majority of the patients (80–93.6%) experience symptom relief following surgical treatment [1, 5, 8, 13, 21, 24, 26]. However, postoperative complications have been reported in 7.6–18.7% of cases, and functional recovery failure has been reported in certain cases despite surgery [1, 8, 20, 26]. Previous studies have identified prolonged symptom duration, preoperative severe deficits, and neurofibromatosis type 2 as significant risk factors associated with poor prognosis [2, 14, 15]. Given the potential for suboptimal outcomes and the tendency of clinicians to recommend conservative management for asymptomatic or mildly symptomatic spinal schwannomas [25], this study aimed to identify prognostic factors associated with poor postoperative outcomes in patients undergoing surgery for solitary spinal schwannomas.

## Methods

### Inclusion criteria and search strategy

A retrospective review was conducted with the clinical data of 72 consecutive patients who underwent direct tumour resection with intraoperative neurophysiological monitoring for symptomatic solitary spinal schwannomas at the Department of Neurosurgery and Orthopaedic Surgery, Saitama Medical Centre, Saitama Medical University, between January 2010 and March 2024. Exclusion criteria were as follows: schwannomas primarily localised in the foraminal and paravertebral regions (e.g., Eden classification type 4) and neurofibromatosis type 2 [6]. A patient with severe preoperative physical impairment due to femoral fractures caused by cervical myelopathy was also excluded. In cases of reoperation for residual tumour and symptomatic recurrence, data from the initial surgery were used. This study was approved by the Institutional Review Board of Saitama Medical Centre, Saitama Medical University (approval number: SOU2024-079). Patient education for study participation was facilitated using an opt-out method on the approved website of the Institutional Review Board. This study complied with the Declaration of Helsinki’s ethical guidelines.

### Data collection

**Fig. 1:**
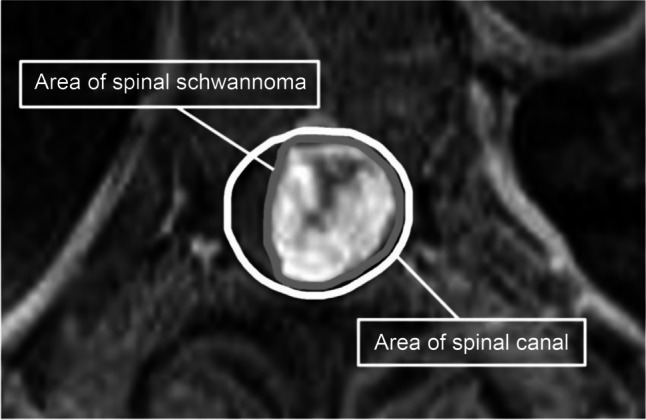
Tumour-occupied ratio based on the axial imaging of the tumour location relative to the spinal canal at the point of maximal spinal cord compression

Patient demographics, including age, sex, body mass index, American Society of Anaesthesiologists physical status classification (ASA-PS), history of diabetes mellitus, and symptom duration, were collected from medical records. Preoperative and postoperative neurological functions were assessed using the Modified McCormick scale (Table 1) [17]. Severe preoperative neurological deficit was defined as a Modified McCormick scale grade of IV–V, and postoperative neurological deficit at final follow-up was defined as a grade of II–V. Moreover, preoperative and postoperative neurogenic pain and bladder/bowel dysfunction (BBD) were evaluated, with persistent dysfunction defined as conditions requiring ongoing pain medication use or urethral catheterisation, respectively. Radiological data, including tumour distribution and dumbbell-shaped appearance, were collected from contrast-enhanced magnetic resonance imaging [3]. The imaging assessments were independently performed by two senior spinal surgeons (SY and KS). The tumour-occupied ratio was calculated from axial images at the point of maximal spinal cord compression using ImageJ software (Fig. 1). The origin nerve root of each tumour was determined based on intraoperative findings. Gross total resection (GTR) was defined as complete microsurgical removal of the tumour confirmed by postoperative imaging. The surgical strategy in our institute prioritises tumour decompression to treat myelopathy rather than extensive resection of the extraspinal portion of a dumbbell-shaped tumour. Additionally, if abnormalities were detected during intraoperative neurophysiological monitoring, preserving neurological function took precedence over complete resection. Instrumented fusion was performed when spinal instability was caused by the tumour or facetectomy. Surgical complications, including postoperative cerebrospinal fluid fistula and vascular injury, were also recorded for each patient.

**Table 1:** Modified McCormick Scale

Grade	Definition
I	Neurologically normal; minimal focal deficit not significantly affecting function; normal gait
II	Mild sensorimotor deficit affecting function; functions and ambulates independently; mild gait difficulty
III	Moderate sensorimotor deficit affecting function; functions and ambulates independently w/external aid; moderate gait difficulty
IV	More severe neurological deficit; requires cane/ brace for ambulation or significant upper-extremity impairment
V	Severe deficit; requires wheelchair or cane/brace w/upper-extremity impairment; usually not independent

### Statistical analysis

Continuous variables were assessed for normality using the Shapiro–Wilk test. The Student’s t-test was applied to normally distributed variables, while the Wilcoxon rank-sum test was used for non-normally distributed variables. Categorical variables were compared using the Chi-square test, with Fisher’s exact test as appropriate. To adjust for potential confounders, multivariable logistic regression analysis was performed, and the overall model fit was assessed using a likelihood ratio test. Data analysis was performed using the JMP 16 software (SAS Institute; Cary, NC, USA), and statistical significance was set at a *p*-value < 0.05.

## Results

### Patient demographics, tumour characteristics, and clinical outcomes

**Fig. 2:**
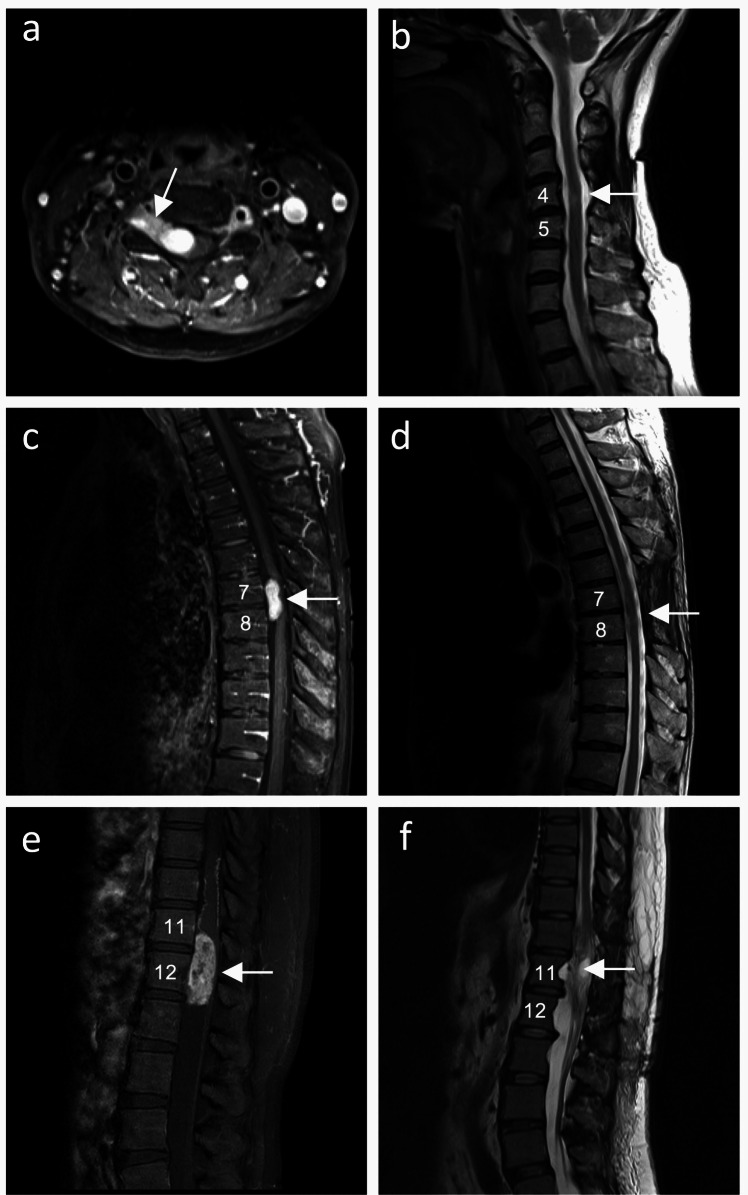
Illustrative cases **A** Preoperative axial gadolinium-enhanced MRI reveals a C5 dumbbell-shaped schwannoma (white arrow). **B** Postoperative sagittal T2-weighted MRI shows that gross total resection was achieved without spinal cord damage (white arrow). **C** A T7 schwannoma localised in the spinal canal is observed preoperatively on sagittal gadolinium-enhanced MRI (white arrow). **D** Postoperative sagittal T2-weighted MRI demonstrates complete resection with no spinal cord damage (white arrow). **E** Preoperative sagittal gadolinium-enhanced MRI showed a thoracic non-dumbbell-shaped schwannoma at T11–12, causing significant spinal cord compression (white arrow). **F** Postoperative T2-weighted MRI revealed areas of partial high intensity within the intramedullary spinal cord (white arrow)

A total of 72 patients who underwent surgical resection for spinal schwannomas were included. Patient demographics and tumour characteristics are summarised in Table 2, and clinical outcomes are presented in Table 3. GTR was achieved in 55 cases (76.4%), and posterior arthrodesis following facetectomy with tumour resection was performed in five cases (6.9%). Two patients (2.8%) with spinal schwannomas originating from the motor root underwent decompressive subtotal resection with intraoperative nerve stimulation, resulting in no postoperative neurological deterioration. Surgical complications were observed in six patients (8.3%), including four cases of cerebrospinal fluid fistula, one case of vertebral artery injury in a patient with a dumbbell-shaped cervical schwannoma, and one case of hemopneumothorax in a patient with a dumbbell-shaped thoracic schwannoma. With a relatively long median follow-up period of 26.4 months, 20 patients (27.8%) exhibited residual neurological deficits. Of these, 12 cases (60.0%) demonstrated neurological recovery but still had residual deficits, 7 cases (35.0%) showed no change in these deficits, and 1 case (5.0%) experienced transient neurological deterioration after surgery with insufficient recovery. Preoperatively, 68.1% of patients experienced neurogenic pain, which decreased to 8.3% with permanent neurogenic pain at final follow-up (*p* = 0.17). Conversely, preoperative BBD significantly improved following tumour resection (9.7% vs. 2.8%; *p* = 0.008). Tumour recurrence was observed in 6 cases (8.3%) during the 26.4-month median follow-up period, all involving incomplete resection. Of these, 3 cases (4.2%) were symptomatic and required additional intervention. Three illustrative cases are presented: one improved case with a cervical dumbbell-shaped tumour, one unchanged case with a thoracic non-dumbbell-shaped tumour, and one worsened case with a lower thoracic non-dumbbell-shaped tumour (Fig. 2). In the first, a 69-year-old woman with a cervical dumbbell-shaped tumour and a Modified McCormick scale grade II presented with a well-circumscribed mass lesion extending into the intervertebral foramen at the C4–5 level on the axial view of the enhanced T1-weighted MRI (Fig. 2A). After undergoing gross total resection, she achieved excellent postoperative recovery without any neurological deficits, showing no evidence of significant spinal cord injury or residual tumour (Fig. 2B). She was discharged home asymptomatic and recurrence-free, with the final follow-up conducted 16 months later. In the second case, an 83-year-old man with a thoracic non-dumbbell-shaped tumour and a Modified McCormick scale grade III exhibited a well-enhanced mass at the T7–8 level on the sagittal view of the MRI (Fig. 2C). Post-surgery, he achieved neurological recovery, though limited to a Modified McCormick scale grade II, despite the complete removal of the tumour as confirmed by postoperative MRI (Fig. 2D). After intensive rehabilitation, he was discharged; however, he exhibited residual gait impairment at the 10-month follow-up, though he did not require a cane. In the third case, a 48-year-old woman with a lower thoracic non-dumbbell-shaped tumour and a Modified McCormick scale grade III presented with a mass filling the spinal canal and severely compressing the spinal cord at T11–12 on MRI (Fig. 2E). Despite undergoing gross total resection, she experienced transient postoperative neurological worsening but ultimately returned to her preoperative McCormick grade III without improvements in bowel-bladder dysfunction (BBD) or neurological pain. Signal changes suggestive of postoperative spinal cord injury were observed on postoperative imaging (Fig. 2F). At the 42-month follow-up, she attended outpatient visits in a wheelchair, managed urination with abdominal pressure, used diapers, and occasionally required painkillers for pain relief.

**Table 2:** Patient demographics and tumour characteristics (*n* = 72)

	Value
Age, median (IQR), year	55.0 (40.8–69.0)
Sex, male, n (%)	41 (56.9)
BMI, mean (SD), kg/m^2^	22.9 (3.2)
ASA-PS, ≥class 3, n (%)	4 (5.6)
History of diabetes mellitus, n (%)	2 (2.8)
Duration of symptoms, median (IQR), month	7.5 (4.0–15.0)
Preoperative severe neurological deficits, modified McCormick scales grade IV–V, n (%)	8 (11.1)
Preoperative spinal pain, n (%)	49 (68.1)
Preoperative bladder/bowel disturbance, n (%)	7 (9.7)
Tumour distribution	
Cervical spine, n (%)	24 (33.3)
Thoracic spine, n (%)	23 (31.9)
Lumbosacral spine, n (%)	25 (34.7)
Tumour-occupied ratio, median (IQR), percent	73.4 (67.3–81.8)
Dumbbell-shaped tumour, n (%)	22 (30.6)
Origin nerve root of tumour, motor root, n (%)	2 (2.8)

*ASA-PS*, American Society of Anaesthesiologists physical status; *BMI*, body mass index;* IQR*, Interquartile range; *SD*, standard deviation

**Table 3:** Clinical outcomes (*n* = 72) IQR, interquartile range

	Value
Gross total resection, n (%)	55 (76.4)
Instrumentation, n (%)	5 (6.9)
Surgical complications, n (%)	6 (8.3)
Residual neurological deficit, modified McCormick scale grade of II–V, n (%)	20 (27.8)
Persistent spinal pain, n (%)	6 (8.3)
Persistent bladder/bowel disturbance, n (%)	2 (2.8)
Symptomatic recurrence requiring reoperation, n (%)	3 (4.2)
Follow-up, median (IQR), month	26.4 (12.0–53.8)

*IQR*, Interquartile range

### Univariable and multivariable analyses

Univariable analysis of clinical factors associated with residual neurological deficits is presented in Table 4. No significant differences were observed in the follow-up period (29.5 months vs. 26.4 months; *p* = 0.48). Regarding tumour characteristics, thoracic spine involvement was more common in patients with residual neurological deficits (65.0% vs. 19.2%; *p* = 0.0003), whereas dumbbell-shaped tumours were less frequent (5.0% vs. 40.4%; *p* = 0.004). No statistically significant association was observed with preoperative severe neurological deficits (*p* = 0.09), although preoperative BBD was significantly more frequent in patients with residual neurological deficits (*p* = 0.02). Surgical complications showed no significant associations with functional outcome (*p* = 0.67). Multivariable logistic regression analysis of the significant clinical factors identified via univariate analysis with a p-value threshold of < 0.1 is summarised in Table 5. The overall model fit was significant (*p* = 0.0002), indicating that the predictors collectively explained the variability in functional outcome. Notably, thoracic spine involvement (odds ratio [OR], 5.03; 95% confidence interval [CI], 1.47–18.6; *p* = 0.01) was identified as a significant risk factor for residual neurological deficits. Conversely, dumbbell-shaped tumours (OR, 0.15; 95% CI, 0.02–1.28; *p* = 0.04) were associated with reduced risk of residual neurological deficits, suggesting that the absence of a dumbbell shape may increase risk. Although severe preoperative neurological deficits and BBD were considered potential confounders related to thoracic spine tumours, the analysis revealed they were not significant independent variables.

**Table 4:** Univariable analysis of clinical factors associated with postoperative residual neurological deficits for spinal schwannomas

	Univariable analysis		
	Residual neurological deficits (*n* = 20)	No neurological deficits (*n* = 52)	*p* value
Age, median (IQR), year	60.5 (47.3–74.5)	54.0 (39.3–67.0)	0.10
Sex, male, n (%)	14 (70.0)	27 (51.9)	0.31
BMI, mean (SD), kg/m^2^	23.2 (4.1)	22.7 (2.8)	0.56
ASA-PS, ≥class 3, n (%)	1 (5.0)	3 (5.8)	1.00
History of diabetes mellitus, n (%)	1 (5.0)	1 (1.9)	0.48
Duration of symptoms, median (IQR), month	9.0 (4.0–19.3)	7.0 (4.0–15.0)	0.56
Preoperative severe neurological deficits, n (%)	4 (20.0)	4 (7.7)	0.09
Preoperative spinal pain, n (%)	14 (70.0)	35 (67.3)	0.83
Preoperative bladder/bowel disturbance, n (%)	5 (25.0)	2 (3.9)	0.02
Tumour distribution, thoracic spine, n (%)	13 (65.0)	10 (19.2)	0.0003
Tumour-occupied ratio, median (IQR), percent	74.0 (70.4–83.8)	72.6 (65.3–81.5)	0.23
Dumbbell-shaped tumour, n (%)	1 (5.0)	21 (40.4)	0.004
Origin nerve root of tumour, motor root, n (%)	1 (5.0)	1 (1.9)	0.48
Gross total resection, n (%)	17 (85.0)	38 (73.1)	0.36
Instrumentation, n (%)	3 (15.0)	2 (3.9)	0.13
Surgical complications, n (%)	2 (10.0)	4 (7.7)	0.67
Follow-up, median (IQR), month	29.5 (10.7–51.7)	26.4 (12.1–59.8)	0.78

*ASA-PS*, American Society of Anaesthesiologists physical status; *BMI*, body mass index; *IQR*, Interquartile range; *SD*, standard deviation

**Table 5:** Logistic regression analysis of clinical factors associated with residual neurological deficits for spinal schwannomas

	Multivariable analysis		
	OR	95% CI	*p* value
Preoperative severe neurological deficits	3.16	0.45–23.2	0.24
Preoperative bladder/bowel disturbance	3.28	0.49–28.9	0.22
Thoracic spine	5.03	1.47–18.6	0.01
Dumbbell-shaped tumour	0.15	0.02–1.28	0.04

*CI*, confidence interval; *OR*, odds ratio

### Poor functional prognosis rate based on tumour distribution and characteristics

**Fig. 3:**
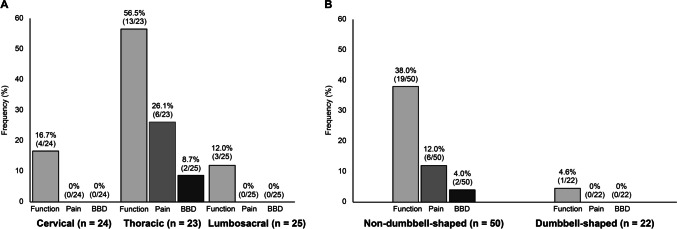
Comparison of poor outcomes based on tumour distribution and characteristics **A** The rate of persistent neurogenic pain, residual neurological deficits, and persistent bladder/bowel disturbances (BBD) on final follow-up were compared based on tumour location using Fisher’s exact test (Function, *p* = 0.001; Pain, *p* = 0.001; BBD, *p* = 0.10). **B** The same outcomes were compared based on whether the tumours were non-dumbbell-shaped or dumbbell-shaped (Function, *p* = 0.004; Pain, *p* = 0.17; BBD, *p* = 1.00)

Comparison of residual deficits of neurological function, neurogenic pain, and BBD was done based on tumour distribution and characteristics (Fig. 3). The incidence of persistent postoperative neurogenic pain was significantly higher in patients with thoracic spinal schwannomas (26.1%; *p* = 0.001). Persistent neurogenic pain was also associated with spinal schwannomas that are not dumbbell-shaped (12.0%), although not reaching statistical significance (*p* = 0.17). On the other hand, permanent BBD was observed only after surgery for thoracic (8.7%) or non-dumbbell-shaped tumours (4.0%).

### Discussion

This retrospective study analysed the clinical and radiological data of 72 patients who underwent surgical resection for solitary spinal schwannomas at our institution. Notably, we identified thoracic spine involvement and the absence of dumbbell-shaped tumour morphology as significant risk factors for residual neurological deficits. These findings enhance surgical decision-making and improve the prediction of postoperative outcomes. They emphasize the importance of advanced surgical management for cases presenting with these risk factors, including expanding the laminectomy range to ensure a safer and more expansive surgical field, employing intraoperative neurophysiological monitoring, and utilizing meticulous surgical techniques.

In this study, thoracic schwannomas were associated with poor postoperative functional recovery compared to cervical and lumbosacral schwannomas. Spinal meningiomas, common in the thoracic spine, share prognostic factors with schwannomas. However, unlike schwannomas, they show no association between tumour location and outcomes [7]. Conversely, upper thoracic schwannomas are consistently linked to poor outcomes [24]. Another study reported that spinal schwannomas involving the thoracolumbar junction were associated with poor prognosis, although they were eligible only for tumours below the thoracolumbar level [28]. The distinct anatomy of the thoracic spinal cord, characterised by poor collateral blood supply, may have contributed to its increased vulnerability to chronic compression and persistent postoperative neurogenic pain [16]. Symptoms of tumour compression can become particularly severe because of the thoracic spine’s kyphotic alignment, narrower canal diameter, and restricted mobility [10]. This is consistent with reports of increased neurological deterioration following surgery for ventral lesions, such as thoracic ossification of the posterior longitudinal ligament [27]. Our findings emphasise the importance of carefully considering the anatomical and vascular characteristics of the thoracic spine upon surgical intervention for thoracic schwannomas, even those originating from non-eloquent nerve roots [11, 12], as these factors may significantly contribute to postoperative neurological deficits compared to other spinal regions.

Our study showed that spinal schwannomas without dumbbell-shaped morphology exhibited a considerable risk of residual neurological deficits following surgery. Previous research has shown that dumbbell-shaped tumours extending beyond the intervertebral foramen along the nerve root pose technical challenges during surgical resection, resulting in postoperative neurological complications [4, 15, 18]. Patients with dumbbell-shaped schwannomas exhibited a higher incidence of cerebrospinal fluid fistula or vascular injury, requiring additional arthrodesis to address facet damage [19, 23]. Such iatrogenic issues are relevant in the backgrounds of these reports. On the other hand, we speculate that our study findings could be attributed to the differences in the frequency of neurological sequelae associated with the residual function of the nerve root of origin [9, 22]. Approximately 7.4% of patients with non-dumbbell-shaped tumours developed persistent neurogenic pain along with poor functional status after surgery, whereas patients with dumbbell-shaped tumours had limited sequelae. This can be attributed to the gradual growth of benign tumours localised within the spinal canal, which also impacts the innervation of neighbouring nerve roots owing to increasing compressive pressure [22]. Furthermore, the mobility of demarcated tumours could have an adverse effect on the spinal cord, increasing the risk of surgical manipulation-related injuries. This clinical awareness could contribute to improved surgical outcomes in patients with spinal schwannomas lacking dumbbell-shaped morphology.

This study has certain limitations. First, its retrospective design, small sample size, and single-centre scope limit the generalisability and statistical power of our findings. Variations in institutional protocols and surgical techniques could introduce biases specific to our centre, reducing external validity. Second, while we utilised the McCormick grading scale for consistency, the absence of standardised neurological evaluation methods across studies complicates direct comparisons with previous studies. Third, the timing of treatment, determined by the attending physician, could introduce variability in patient outcomes, particularly in cases of delayed intervention. Fourth, surgeries performed by five different surgeons with varying levels of expertise may have influenced differences in surgical techniques and patient outcomes. Furthermore, variations in follow-up duration among patients may have impacted the assessment of long-term outcomes, and incomplete data from some patients further limit the comprehensiveness of our analysis.

## Conclusions

Our study identified thoracic spine involvement and tumours without dumbbell-shaped morphology as significant risk factors for residual neurological deficits in patients undergoing surgical treatment for sporadic spinal schwannomas. A comprehensive understanding of tumour distribution and characteristics can aid in thorough preoperative evaluations, fostering informed decision-making and optimised surgical management, thereby improving patient prognosis while acknowledging the multifactorial nature of patient recovery after surgery.

## Data Availability

No datasets were generated or analysed during the current study.

## References

[1] Alvarez-Crespo DJ, Conlon M, Kazim SF et al (2024) Clinical characteristics and surgical outcomes of 2542 patients with spinal schwannomas: a systematic review and meta-analysis. World Neurosurg 182:165–183e1 38006933 10.1016/j.wneu.2023.11.090

[2] Ando K, Kobayashi K, Nakashima H, Machino M, Ito S, Kanbara S, Inoue T, Segi N, Koshimizu H, Imagama S (2020) Surgical outcomes and factors related to postoperative motor and sensory deficits in resection for 244 cases of spinal schwannoma. J Clin Neurosci 81:6–11 33222969 10.1016/j.jocn.2020.09.025

[3] Beks JW, Penning L, van der Zwaag P, Ebels EJ (1966) Dumbbell tumours in the spinal canal. Psychiatr Neurol Neurochir 69(6):399–410 5971456

[4] Celli P (2002) Treatment of relevant nerve roots involved in nerve sheath tumors. Remov or Preservation? Neurosurg 51(3):684–692 12188946

[5] Conti P, Pansini G, Mouchaty H, Capuano C, Conti R (2004) Spinal neurinomas: retrospective analysis and long-term outcome of 179 consecutively operated cases and review of the literature. Surg Neurol 61(1):34–43 discussion 44 14706374 10.1016/s0090-3019(03)00537-8

[6] Eden K (2005) The dumb-bell tumours of the spine. Br J Surg 28(112):549–570

[7] El-Hajj VG, Pettersson-Segerlind J, Fletcher-Sandersjöö A, Edström E, Elmi-Terander A (2022) Current knowledge on spinal meningiomas—surgical treatment, complications, and outcomes: a systematic review and meta-analysis (part 2). Cancers (Basel) 14(24):6221 36551706 10.3390/cancers14246221 PMC9777510

[8] Hohenberger C, Hinterleitner J, Schmidt N-O, Doenitz C, Zeman F, Schebesch K-M (2020) Neurological outcome after resection of spinal schwannoma. Clin Neurol Neurosurg 198(106127):106127 32768692 10.1016/j.clineuro.2020.106127

[9] Jinnai T, Hoshimaru M, Koyama T (2005) Clinical characteristics of spinal nerve sheath tumors: analysis of 149 cases. Neurosurgery 56(3):510–515 15730576 10.1227/01.neu.0000153752.59565.bb

[10] Kang MS, Park JY, Chin DK, Kim KH, Kuh SU, Kim KS, Cho YE (2012) A PET/CT-based morphometric study of spinal canal in Korean young adults: Anteroposterior diameter from cervical vertebra to sacrum. Korean J Spine 9(3):165 25983809 10.14245/kjs.2012.9.3.165 PMC4430996

[11] Kim P, Ebersold MJ, Onofrio BM, Quast LM (1989) Surgery of spinal nerve schwannoma: risk of neurological deficit after resection of involved root. J Neurosurg 71(6):810–814 2585070 10.3171/jns.1989.71.6.0810

[12] Klekamp J, Samii M (1998) Surgery of spinal nerve sheath tumors with special reference to neurofibromatosis. Neurosurgery 42(2):279–289 9482178 10.1097/00006123-199802000-00042

[13] Lenzi J, Anichini G, Landi A, Piciocchi A, Passacantilli E, Pedace F, Delfini R, Santoro A (2017) Spinal nerves schwannomas: experience on 367 cases—historic overview on how clinical, radiological, and surgical practices have changed over a course of 60 years. Neurol Res Int 2017:1–12 10.1155/2017/3568359 PMC5624174 29075532

[14] Li P, Zhao F, Zhang J, Wang Z, Wang X, Wang B, Yang Z, Yang J, Gao Z, Liu P (2016) Clinical features of spinal schwannomas in 65 patients with schwannomatosis compared with 831 with solitary schwannomas and 102 with neurofibromatosis type 2: a retrospective study at a single institution. J Neurosurg Spine 24(1):145–154 26407091 10.3171/2015.3.SPINE141145

[15] Liu Z, Xu Z, Shen J et al (2023) Scoring model to predict postoperative neurological deterioration in spinal schwannoma. Front Oncol 13:1086299 36998448 10.3389/fonc.2023.1086299 PMC10043432

[16] Martirosyan NL, Feuerstein JS, Theodore N, Cavalcanti DD, Spetzler RF, Preul MC (2011) Blood supply and vascular reactivity of the spinal cord under normal and pathological conditions: a review. J Neurosurg Spine 15(3):238–251 21663407 10.3171/2011.4.SPINE10543

[17] McCormick PC, Torres R, Post KD, Stein BM (1990) Intramedullary ependymoma of the spinal cord. J Neurosurg 72(4):523–532 2319309 10.3171/jns.1990.72.4.0523

[18] Nakamura M, Iwanami A, Tsuji O, Hosogane N, Tsuji T, Ishii K, Toyama Y, Chiba K, Matsumoto M, Watanabe K (2013) Long-term surgical outcomes of cervical dumbbell neurinomas. J Orthop Sci 18(1):8–13 22948961 10.1007/s00776-012-0300-2

[19] Safaee MM, Lyon R, Barbaro NM, Chou D, Mummaneni PV, Weinstein PR, Chin CT, Tihan T, Ames CP (2017) Neurological outcomes and surgical complications in 221 spinal nerve sheath tumors. J Neurosurg Spine 26(1):103–111 27472744 10.3171/2016.5.SPINE15974

[20] Safavi-Abbasi S, Senoglu M, Theodore N, Workman RK, Gharabaghi A, Feiz-Erfan I, Spetzler RF, Sonntag VKH (2008) Microsurgical management of spinal schwannomas: evaluation of 128 cases. J Neurosurg Spine 9(1):40–47 18590409 10.3171/SPI/2008/9/7/040

[21] Satoh N, Koizumi M, Takeshima T, Iida J, Matsumori H, Tanaka Y, Ueda Y, Shigematsu K, Shigematsu H (2011) Assessment of pure single nerve root resection in the treatment of spinal schwannoma: focus on solitary spinal schwannomas located below the thoracolumbar junction. J Orthop Sci 16(2):148–155 21311929 10.1007/s00776-011-0032-8

[22] Schultheiss R, Gullotta G (1993) Resection of relevant nerve roots in surgery of spinal neurinomas without persisting neurological deficit. Acta Neurochir (Wien) 122(1–2):91–96 8333314 10.1007/BF01446992

[23] Sebai MA, Kerezoudis P, Alvi MA, Yoon JW, Spinner RJ, Bydon M (2019) Need for arthrodesis following facetectomy for spinal peripheral nerve sheath tumors: an institutional experience and review of the current literature. J Neurosurg Spine 31(1):112–122 30952137 10.3171/2019.1.SPINE181057

[24] Subramanian A, Nair BR, Rajshekhar V (2021) Functional outcomes and temporal profile of recovery in patients with intradural extramedullary spinal cord tumors with poor Nurick grade. World Neurosurg 146:e691–e700 33171318 10.1016/j.wneu.2020.10.168

[25] Sun I, Pamir MN (2017) Non-syndromic spinal schwannomas: a novel classification. Front Neurol 8:318 28769861 10.3389/fneur.2017.00318 PMC5511849

[26] Xin Z, Orazmyradov B, Li J et al (2020) A novel classification and its clinical significance in spinal schwannoma based on the membranous hierarchy. Neurosurgery 87(6):1206–1222 32691825 10.1093/neuros/nyaa272

[27] Xu N, Yu M, Liu X, Sun C, Chen Z, Liu Z (2017) A systematic review of complications in thoracic spine surgery for ossification of the posterior longitudinal ligament. Eur Spine J 26(7):1803–1809 26179087 10.1007/s00586-015-4097-5

[28] Zou F, Guan Y, Jiang J, Lu F, Chen W, Xia X, Wang L, Ma X (2016) Factors affecting postoperative neurological deficits after nerve root resection for the treatment of spinal intradural schwannomas. Spine (Phila Pa 1976) 41(5):384–389 26919412 10.1097/BRS.0000000000001248

